# Inhibition of sarcoplasmic Ca^2+^-ATPase increases caffeine- and halothane-induced contractures in muscle bundles of malignant hyperthermia susceptible and healthy individuals

**DOI:** 10.1186/1471-2253-5-8

**Published:** 2005-06-09

**Authors:** Frank Schuster, Rainer Müller, Edmund Hartung, Norbert Roewer, Martin Anetseder

**Affiliations:** 1 Department of Anaesthesiology, University of Wuerzburg Oberduerrbacher Strasse 6, 97080 Wuerzburg, Germany; 2 Department of Anaesthesiology and Intensive Care, Hospital of Stralsund, 18410 Stralsund, Germany

## Abstract

**Background:**

Malignant hyperthermia (MH) is triggered by halogenated anaesthetics and depolarising muscle relaxants, leading to an uncontrolled hypermetabolic state of skeletal muscle. An uncontrolled sarcoplasmic Ca^2+ ^release is mediated via the ryanodine receptor. A compensatory mechanism of increased sarcoplasmic Ca^2+^-ATPase activity was described in pigs and in transfected cell lines. We hypothesized that inhibition of Ca^2+ ^reuptake via the sarcoplasmic Ca^2+^-ATPase (SERCA) enhances halothane- and caffeine-induced muscle contractures in MH susceptible more than in non-susceptible skeletal muscle.

**Methods:**

With informed consent, surplus muscle bundles of 7 MHS (susceptible), 7 MHE (equivocal) and 16 MHN (non-susceptible) classified patients were mounted to an isometric force transducer, electrically stimulated, preloaded and equilibrated. Following 15 min incubation with cyclopiazonic acid (CPA) 25 μM, the European MH standard in-vitro-contracture test protocol with caffeine (0.5; 1; 1.5; 2; 3; 4 mM) and halothane (0.11; 0.22; 0.44; 0.66 mM) was performed. Data as median and quartiles; Friedman- and Wilcoxon-test for differences with and without CPA; p < 0.05.

**Results:**

Initial length, weight, maximum twitch height, predrug resting tension and predrug twitch height of muscle bundles did not differ between groups. CPA increased halothane- and caffeine-induced contractures significantly. This increase was more pronounced in MHS and MHE than in MHN muscle bundles.

**Conclusion:**

Inhibition of the SERCA activity by CPA enhances halothane- and caffeine-induced contractures especially in MHS and MHE skeletal muscle and may help for the diagnostic assignment of MH susceptibility. The status of SERCA activity may play a significant but so far unknown role in the genesis of malignant hyperthermia.

## Background

In skeletal muscle, the action potential passes along the surface membrane of the muscle fibre into the transverse tubular system. Depolarisation of the voltage sensitive dihydropyridine receptor leads to an opening of the ryanodine receptor in the nearby sarcoplasmic reticulum (SR). Sarcoplasmic calcium (Ca^2+^) release via the ryanodine receptor raises cytosolic Ca^2+ ^and activates muscle contraction. Energy-dependent Ca^2+ ^reuptake into the SR is caused by the SR Ca^2+^-ATPase (SERCA) and enables skeletal muscle relaxation [1]. In individuals susceptible to the autosomal dominant skeletal muscle disorder malignant hyperthermia (MH), electro-mechanical coupling is disturbed. Due to MH-associated mutations in the ryanodine receptor, triggering agents such as halogenated anaesthetics cause an excessive Ca^2+ ^release from the SR resulting in intracellular hypermetabolism, increased mitochondrial energy-turnover and metabolic failure with a deficiency of adenosine-triphosphate [2]. This may also lead to energetic exhaustion of the SERCA, the main transporter for Ca^2+ ^ions across the sarcoplasmic membrane. Cyctosolic Ca^2+ ^concentration is determined by sarcoplasmic Ca^2+ ^release and it's reuptake via the SERCA [3]. The mycotoxin cyclopiazonic acid (CPA) is a selective inhibitor of SR Ca^2+ ^reuptake [4] that has been used previously to study SERCA in different tissues [5,6].

We hypothesized that in skeletal muscle, preincubation with CPA enhances halothane- and caffeine-induced contractures in MH susceptible (MHS) more than in non-susceptible (MHN) skeletal muscle.

## Methods

Muscle bundles of 30 patients undergoing a diagnostic in-vitro contracture test (IVCT) were investigated to detect MH susceptibility. With informed consent, surplus muscle bundles were studied by the same IVCT protocol following SERCA inhibition by CPA.

### Muscle biopsy

A muscle biopsy of the vastus lateralis muscle was performed following a femoral nerve block. Muscle bundles were immediately placed in carboxygenated (95% oxygen, 5% carbon dioxide) Krebs-Ringer's solution (NaCl 118.1 mM; KCl 3.4 mM; CaCl_2 _2.5 mM; MgSO_4 _0.8 mM; KH_2_PO_4 _1.2 mM; NaHCO_3 _25.0 mM; Glucose 11.1 mM) and transported to the laboratory.

### Standard IVCT

In brief, after length and wet weight of each muscle bundle was measured, single muscle strips were mounted vertically in the experimental bath perfused with carboxygenated Krebs-Ringer's solution at 37°C, fixed to an isometric force transducer (Lectromed Type 4150, UK) and stimulated electrically with a supramaximal square wave stimulus at 1 ms duration and a frequency of 0.2 Hz (Hugo-Sachs-Elektronik, Type 215/I, Germany). Resting tension and twitch height of the muscle strips were recorded continuously by a digital recording system (MusCo, RS BioMed, Germany). After equilibration, caffeine (Sigma Chemicals, Germany) respectively halothane (Abott, Germany) were given at increasing concentrations of 0.5; 1; 1.5; 2; 3; 4; and 32 mM respectively 0.11; 0.22; 0.44 and 0.66 mM at 3 min intervals. A contracture < 2 mN at caffeine 2 mM and halothane 0.44 mM was classified MHN. A stronger contracture following only one of both drugs lead to the diagnosis MH equivocal (MHE). If both drugs developed a significant contracture the patient was assigned as MHS. Investigations were performed within 5 hours after muscle biopsy [7].

### CPA-IVCT

A modified contracture test was carried out studying the drug CPA (M = 336.38 g mol^-1^) that was prepared in a stock solution at 2.5 mM dissolved in dimethylsulphoxide 0,5% (DMSO) (all Sigma Chemicals, Germany). Following equilibration as described above, muscle bundles were incubated with CPA 25 μM for 15 min. The contracture test was then carried out as described above.

### Statistics

Data are shown as median and quartiles. IVCT results of skeletel muscle contractures with CPA were statistically evaluated in comparison to the results without CPA by using the Friedman- and Wilcoxon-test for differences with and without CPA. p < 0.05 was considered significant.

## Results

Thirty patients, 9 female and 21 male, with a mean age of 28 (15 – 32) years and a mean weight of 74 (62 – 87) kg were studied. 7 patients were classified as MHS, 7 as MHEh (susceptible only for halothane) and 16 as MHN according to the criteria of the European Malignant Hyperthermia group diagnostic protocol. In every patient, an additional IVCT with CPA was performed. Muscle bundles used for the IVCT and CPA-IVCT did not differ regarding to length, weight, maximum twitch height, predrug resting tension and predrug twitch height (Table 1).

**Table 1:** Biometric data of muscle bundles used for the In-vitro Contracture-Test without (IVCT) and with preincubation with cyclopiazonic acid (CPA-IVCT); median and quartiles.

	**IVCT**	**CPA-IVCT**
**Length (mm)**	18 (16 – 20)	18 (15 – 20)
**Weight (mg)**	220 (190 – 233)	205 (180 – 240)
**Maximum twitch height (mN)**	24 (22 – 27)	25 (23 – 27)
**Predrug resting tension (mN)**	11 (9 – 14)	11 (8 – 14)
**Predrug twitch height (mN)**	57 (37 – 75)	42 (16 – 82)

In the caffeine contracture test, prior incubation with CPA resulted in significant higher contractures compared to the diagnostic IVCT in the MHS and MHEh group (Table 2). At the diagnostic threshold dose of caffeine 2 mM, MHS muscles developed significantly higher contractures with 32 (25 – 38) mN following preincubation with CPA vs. 8 (4 – 12) mN without CPA. In the MHEh group CPA preincubation lead to significantly higher contractures with 12 (11 – 27) mN vs. 1 (0 – 1) mN without CPA, while the contractures of MHN muscle bundles did not differ with or without CPA.

**Table 2:** Caffeine-induced contractures with and without preincubation by cyclopiazonic acid 25 μM (CPA); median and quartile; * p < 0.05 for differences with CPA and without CPA.

**Caffeine [mM]**	**0.5**	**1**	**1.5**	**2**	**3**	**4**	**32**
**MHS [mN]**	1 (0 – 1)	1 (1 – 1)	3 (1 – 6)	8 (4 – 12)	20 (15 – 31)	21 (14 – 35)	171 (136 – 137)
**MHS_CPA _[mN]**	3 (2 – 8)*	14 (11 – 31)*	27 (17 – 47)*	32 (25 – 38)*	31 (27 – 37)	34 (29 – 38)	131 (108 – 191)

**MHEh [mN]**	1 (1 – 2)	1 (1 – 1)	0 (0 – 0)	1 (0 – 1)	2 (1 – 3)	4 (3 – 7)	127 (108 – 190)
**MHEh_CPA _[mN]**	0 (0 – 1)	3 (0 – 5)	7 (3 – 19)*	12 (11 – 27)*	31 (29 – 37)*	46 (31 – 47)*	199 (156 – 227)

**MHN [mN]**	1 (0 – 1)	1 (0 – 1)	1 (1 – 1)	1 (0 – 1)	1 (0 – 1)	1 (0 – 2)	158 (108 – 176)
**MHN_CPA _[mN]**	2 (1 – 2)	2 (1 – 2)	1 (1 – 5)	1 (1 – 6)	4 (1 – 21)*	17 (4 – 27)*	167 (153 – 180)

At halothane 0.44 mM, CPA preincubation increased contractures of MHS and MHEh muscle bundles significantly to 59 (33 – 73) mN respectively 45 (24 – 55) mN compared to standard IVCT conditions with 20 (16 – 26) mN respectively 4 (2 – 4) mN. In addition, in the MHN group at halothane 0.44 mM contractures were significantly increased by CPA preincubation to 16 (4 – 34) mN vs. 1 (1 – 1) mN without CPA (Table 3).

**Table 3:** Halothane-induced contractures with and without cyclopiazonic acid 25 μM (CPA) pre-incubation; median and quartile; * p < 0.05 for differences between IVCT and CPA-IVCT.

**Halothane [mM]**	**0.11**	**0.22**	**0.44**	**0.66**
**MHS [mN]**	5 (2 – 6)	14 (13 – 20)	20 (16 – 26)	19 (11 – 24)
**MHS_CPA _[mN]**	40 (26 – 58)*	52 (29 – 76)*	59 (33 – 73)*	48 (30 – 57)*

**MHEh [mN]**	0 (0 – 0)	(0 – 1)	4 (2 – 4)	3 (2 – 4)
**MHEh_CPA _[mN]**	7 (1 – 11)*	25 (13 – 39)*	45 (24 – 55)*	45 (25 – 48)*

**MHN [mN]**	1 (0 – 2)	1 (0 – 1)	1 (1 – 1)	1 (0 – 1)
**MHN_CPA _[mN]**	1 (0 – 3)	2 (0 – 25)	16 (4 – 34)*	20 (9–31)*

## Discussion

In MH uncontrolled SR Ca^2+ ^release, caused by MH associated mutations mainly in the ryanodine receptor gene, is widely accepted as the underlying pathophysiological mechanism of hypermetabolism [8]. However, the detection of a mutation in the alpha 1-subunit of the voltage sensitive dihydropyridine receptor in a French MH family suggests a more complex pathogenesis of MH [9]. According to the unique mechanism of intracellular Ca^2+ ^cycling that induces contraction and relaxation in vertebrate skeletal muscle, sarcoplasmic Ca^2+ ^release and sarcoplasmic Ca^2+ ^reuptake determine the mainstays of Ca^2+ ^regulation. Undoubtly, an altered SR Ca^2+ ^release plays a crucial role in the development of MH. However, it is completely unclear why many MHS individuals may suffer from MH only after several uneventful exposures to trigger agents during anaesthesia. Several modulating factors have been postulated to modulate cytosolic Ca^2+ ^concentrations, e.g. magnesium [10], sympathetic activity [11], temperature [12], volatile anesthetics [13] or channel's redox state [14]. While SR Ca^2+ ^release was extensively studied in MH [15], the impact of an altered SR Ca^2+ ^reuptake on the pathogenesis of MH by intrinsic or extrinsic factors is poorly understood. Theoretically, a reduced activity of the skeletal muscular SERCA type 1 may result in an elevated cytosolic Ca^2+ ^level due to a persistent slow Ca^2+ ^efflux out of the SR that is otherwise balanced by reuptake [16]. A critical threshold of cytosolic Ca^2+ ^may then be exceeded and may lead to contracture development in vitro and to the MH syndrome in susceptible patients. Interestingly, CPA alone did not induce skeletal muscle contractures at 25 μM [17]. We assume that in our study SERCA was inhibited almost completely, since CPA 10 μM reduced the SERCA activity approximately by 70% in frog skinned fibres [16] and nearly by 100% in rat skinned fibres [18].

In the presented study, CPA preincubation lead to a high variability of halothane- respectively caffeine-induced contractures especially in the MHS and MHEh group, despite SERCA distribution does not differ between MHS and MHN muscle [19]. Interestingly, the response of MHEh muscle bundles to caffeine was enhanced by CPA preincubation. However, at this stage, our results do not suggest CPA as an alternative approach to improve differentiation of MHE from MHN respectively MHS individuals.

Ca^2+ ^uptake capacity and SERCA activity was found to be significantly increased in MHS pigs [20] and in HEK-293 cells transfected by MH mutants [21] but was described to be lower in MHS muscle compared to normal human skeletal muscle [22]. Since a leaky ryanodine receptor in MHS individuals may lead to increased cytosolic calcium, it looks feasible that SR-Ca-ATPase may be upregulated by a compensatory mechanism.

Another option is that CPA itself modulates directly the effect of the trigger agent. This is less likely since halothane and caffeine do have different binding sites at the sarcoplasmic membrane [23].

The role of a reduced SERCA activity in the pathogenesis of Brody's disease, a skeletal muscular myopathy, is well known and characterized by painless muscle cramping and exercise-induced muscle stiffness linked to a mutation in the gene encoding SERCA [24,25]. The left-shift of the dose-response curve for halothane- and caffeine-induced contractures following inhibition of the sarcoplasmic Ca^2+ ^reuptake by CPA points out the essential part of SERCAs in the regulation of cytoplasmic Ca^2+^. We believe this may be an explanation why some MH susceptible patients develop a MH crisis while others never or only after several trigger exposures suffer from MH despite a proven in vitro susceptibility. In this context, an altered activity of SERCA due to intrinsic or extrinsic factors may play a crucial role in the evolution of MH.

## Conclusion

The present study demonstrates that CPA preincubation enhances halothane- and caffeine-induced muscle contractures in the IVCT of MHS, MHEh more than in MHN patients.

Modulation of SERCA may play a significant role in the development of malignant hyperthermia. Patients with a high activity may compensate an increased Ca^2+ ^release or leakage from the SR while patients with a low activity of the SERCA do not. Further investigations with focus on extrinsic and intrinsic factors that modulate SERCA activity may be helpful to understand why MH patients may have had several anaesthesias including trigger agents without a significant reaction while developing a fulminate MH crisis at another occasion.

## Abbreviations

Ca^2+ ^Calcium

CPA Cyclopiazonic acid

IVCT In-Vitro Contracture Test

MH Malignant hyperthermia

MHEh Malignant hyperthermia equivocal; susceptible only for halothane

MHN Malignant hyperthermia non-susceptible

MHS Malignant hyperthermia susceptible

SERCA Sarcoplasmic calcium adenosine triphosphatase

SR Sarcoplasmic reticulum

## Authors' contributions

FS collected and analysed the data and drafted the manuscript. RM collected data and performed the statistical analysis. EH conceived the study. NR participated in the design of the study. MA designed the study protocol, accompanied the data acquisition and helped writing the manuscript. All authors read and approved the final manuscript.

## Pre-publication history

The pre-publication history for this paper can be accessed here:


